# Characterisation of Expression the Arginine Pathway Enzymes in Childhood Brain Tumours to Determine Susceptibility to Therapeutic Arginine Depletion

**DOI:** 10.1155/2022/9008685

**Published:** 2022-06-22

**Authors:** Eleanor Bishop, Monika Dimitrova, Alexander Froggatt, Maria Estevez-Cebrero, Lisa C. D. Storer, Francis Mussai, Simon Paine, Richard G. Grundy, Madhumita Dandapani

**Affiliations:** ^1^Childrens Brain Tumour Research Centre, School of Medicine, University of Nottingham, UK; ^2^Institute of Immunology and Immunotherapy, University of Birmingham, Birmingham, UK; ^3^Nottingham University Hospitals NHS Trust, Nottingham, UK

## Abstract

Despite significant improvements in treatment and survival in paediatric cancers, outcomes for children with brain tumours remain poor. Novel therapeutic approaches are needed to improve survival and quality of survival. Extracellular arginine dependency (auxotrophy) has been recognised in several tumours as a potential therapeutic target. This dependency is due to the inability of cancer cells to recycle or synthesise intracellular arginine through the urea cycle pathway compared to normal cells. Whilst adult glioblastoma exhibits this dependency, the expression of the arginine pathway enzymes has not been delineated in paediatric brain tumours. We used immunohistochemical (IHC) methods to stain for arginine pathway enzymes in paediatric high-grade glioma (pHGG), low-grade glioma (pLGG), ependymoma (EPN), and medulloblastoma (MB) tumour tissue microarrays (TMAs). The antibodies detected protein expression of the metaboliser arginase (Arg1 and Arg2); recycling enzymes ornithine transcarbamoylase (OTC), argininosuccinate synthetase (ASS1), and argininosuccinate lyase (ASL); and the transporter SLC7A1. Deficiency of OTC, ASS1, and ASL was seen in 87.5%, 94%, and 79% of pHGG samples, respectively, consistent with an auxotrophic signature. Similar result was obtained in pLGG with 96%, 93%, and 91% of tumours being deficient in ASL, ASS1, and OTC, respectively. 79%, 88%, and 85% of MB cases were ASL, ASS1, and OTC deficient whilst ASL and OTC were deficient in 57% and 91% of EPN samples. All tumour types highly expressed SLC7A1 and Arginase, with Arg2 being the main isoform, demonstrating that they could transport and utilise arginine. Our results show that pHGG, pLGG, EPN, and MB demonstrate arginine auxotrophy based on protein expression and are likely to be susceptible to arginine depletion. Pegylated arginase (BCT-100) is currently in phase I/II trials in relapsed pHGG. Our results suggest that therapeutic arginine depletion may also be useful in other tumour types and IHC analysis of patient tumour samples could help identify patients likely to benefit from this treatment.

## 1. Introduction

Paediatric central nervous system (CNS) tumours are the commonest solid tumour in childhood and account for around 25% of paediatric cancers [1]. They are the leading cause of cancer-related death in children [2]. Despite improvements in the survival and cure rates of other childhood cancers, the prognosis for several types of brain tumour remains poor [1]. Treatment-related morbidity is high with significant late effects in survivors [3]. There is therefore a need to develop kinder, effective treatments to increase survival and reduce late effects.

One of the hallmarks of cancer is the ability of the tumour cell to alter its cellular machinery to promote growth and survival [4]. One such adaptation is the Warburg effect, reported almost a century ago, wherein the cancer cell derives its energy by anaerobic glycolysis, even in the presence of oxygen [5]. Targeting the metabolic machinery of the cell through various means has since been pursued to develop treatments for cancer. The folate antagonist drug methotrexate is one such example [6].

In recent years, there is increasing evidence that cancer cells also rewire the metabolism of amino acids [7, 8]. Amino acids are the building blocks of protein and also serve as neurotransmitters and alternative substrates for glycolysis and are required for nucleotide synthesis [7]. Tumour cells often exhibit specific metabolic dependencies, such as the increased requirement of certain amino acids to fuel their growth or to modulate the immune response to the tumour [9]. Exploiting unique aspects of tumour metabolism aims to selectively “attack” those tumour cells, effectively avoiding normal tissue toxicity [10]. The concept of targeting amino acid metabolism has been studied for many decades, and the most successful example to date is the depletion of asparagine in the treatment of acute lymphoblastic leukaemia [11].

Arginine is a semiessential amino acid that plays a crucial role in cellular metabolism and homeostasis. It is central to the urea cycle and is a key precursor for polyamine and nitric oxide (NO) production [12]. During periods of stress, such as the hostile tumour microenvironment, cells are unable to meet metabolic demands for arginine. In such conditions, arginine functions as an essential amino acid, which is critical for cell survival, growth, and other hallmarks of cancer [13] The depletion of semiessential amino acids could prove lethal to those cells, thus, further delineating that the role of arginine in cancer could provide novel therapeutic opportunity.

Cells obtain arginine from two key sources: exogenous arginine from dietary protein degradation and *de novo* synthesis inside the cell [14]. Extracellular arginine is transported into the cell via the SLC (solute carrier family) membrane transporters, notably SLC7A1, and is metabolised by a number of enzymes including arginase (Arg), of which there are two human isoforms Arg1 and Arg2 [15, 16]. Arginine can also be obtained by the recycling of precursors: ornithine and citrulline, via the urea cycle. This is achieved through the complement of anabolic enzymes: ornithine carbamoyl transferase (OTC), argininosuccinate synthase 1 (ASS1), and argininosuccinate lyase (ASL) [15].

Cells deficient in any one of the three recycling enzymes are solely dependent upon extracellular sources of arginine, a state termed arginine auxotrophism [17]. This presents a novel therapeutic opportunity, whereby reduction of arginine below a threshold level results in cytotoxicity [15]. A growing number of tumour types have been described as arginine auxotrophic, due to variable loss of ASS1 [13]. These include renal cancer, prostate cancer, and notably two of the most aggressive solid malignancies: hepatocellular carcinoma (HCC) and malignant melanoma [13]. ASS1 has been described as the rate-limiting enzyme in arginine resynthesis; thus, arginine depletion has been explored as a strategy in these ASS1-deficient tumours [18].

It is also important to consider the status of OTC, as its deficiency would prevent the use of ornithine in the recovery of arginine [18]. Lack of OTC in melanoma cells correlates with sensitivity to arginine depletion [19]. Additional antitumour effects have also been seen in HCC, where ASS is present, but there is a reduction in OTC expression [13]. Deficiency in OTC has the advantage that ornithine rarely substitutes for arginine, whereas citrulline often can [20]. The absence of OTC in gliomas would suggest that these tumours primarily use ornithine for alternative functions opposed to arginine regeneration [21].

In comparison to tumour tissue, normal cells often have fully functioning ASS and OTC and, thus, can resynthesise arginine from ornithine and citrulline. This resultant disparity between normal and tumour tissues represents a therapeutic window to selectively target tumour cells [15]. Arginine depletion has been shown to significantly reduce the viability of several different types of tumour including AML blasts, neuroblastoma, and glioblastoma [22–24] and has moved into the clinical setting following the development of two novel drugs, arginine deiminase (ADI) and BCT-100 (PEGylated human recombinant Arg1)[24, 33]. There is currently an ongoing early-phase clinical trial of BCT-100 in paediatric cancers (NCT03455140).

To date, the status of arginine auxotrophy has not been fully characterised in any paediatric brain tumour tissue at the protein level. However, analysis of genomic data from the R2 platform (http://r2.amc.nl/) suggests that several brain tumours, including gliomas, are auxotrophic for arginine, with cells deficient in both OTC and ASS [25]. Additionally, two high-grade glioma (HGG) cell lines (GO-C-CCM and U-870-MG) were shown to require arginine for tumourigenic growth and both died within 5 days of cell culture without arginine [26]. The immediate downstream metabolite of arginine is polyamine, which contributes to the pathogenesis of glioblastomas, and unsurprisingly, arginine depletion reduces the invasiveness of glioblastoma cell lines [27]. This paper characterises the expression of arginine pathway enzymes in several paediatric CNS tumours, namely, high-grade glioma (pHGG), paediatric low-grade glioma (LGG), ependymoma (EPN), and medulloblastoma (MB).

## 2. Material and Methods

### 2.1. Immunohistochemistry

Immunohistochemistry (IHC) was performed on tissue microarrays (TMAs), created within the Children's Brain Tumour Research Centre (CBTRC). These have been collected and stored with the appropriate consents and approvals.

Sections were cut five *μ*m thick from each TMA block and mounted onto a glass microscope slide coated with 3-aminopropyltriethoxysilane (APES). Paraffin wax was removed by immersion of the slides in xylene for 15 min. Rehydration of the slides was achieved by passing through 100% ethanol followed by 95% ethanol, for 10 min each, and finishing in tap water. Heat-mediated antigen retrieval was performed by incubating the slides in sodium citrate buffer (pH 6) for 40 min in a steamer (for OTC, ASS1, ASL, ARG1, and ARG2) or for 5 min in a pressure cooker (for SLC7A1). Slides were then blocked with 20% normal goat serum in PBS for 20 min followed by Peroxidase-Blocking Solution (DAKO-Agilent, S202386-2) for 5 min. Afterwards, the primary antibodies diluted in Antibody Diluent (DAKO-Agilent, S080983-2) were applied on the positive control tissue and test slides at the appropriate dilutions, previously optimised and confirmed by neuropathologists from Nottingham University Hospitals. Staining was performed with anti-OTC (HPA000243) at 1 : 2500 and anti-ARG1 (HPA003595) at 1 : 5000 using liver as a control; anti-ASS1 (HPA020934) at 1 : 750, anti-ASL (HPA016646) at 1 : 2500, and anti-ARG1 (HPA000663) at 1 : 200 using kidney as a control; and anti-SCL7A1 (HPA039721) at 1 : 20 using small bowel tissue as a control (Table 1). All antibodies were purchased from Sigma-Aldrich. Incubations were conducted for 1 h at room temperature for all primary antibodies, except for anti-ARG2 and SCL7A1 that were performed overnight at 4°C. After a wash with PBS for 5 min, the secondary antibody (DAKO-Agilent, K500711-2) was applied on the sections and incubated for 30 min at room temperature. Then, another wash with PBS followed and the slides were incubated with DAB solution (DAKO-Agilent, K500711-2) for 15 min and rinsed with water. Specimens were then counterstained with Gill's 3 Haematoxylin solution (TSC Bioscientific) for 10 sec, rinsed in water, and immersed in Lithium Carbonate (Sigma-Aldrich) for further 10 sec. Dehydration of the tissues was achieved by going through 95% ethanol, followed by 100% ethanol and finishing in xylene. Slides were mounted with DPX medium (Sigma-Aldrich) onto a coverslip for microscopy analysis.

### 2.2. Scanning and Scoring

**Figure 1:**
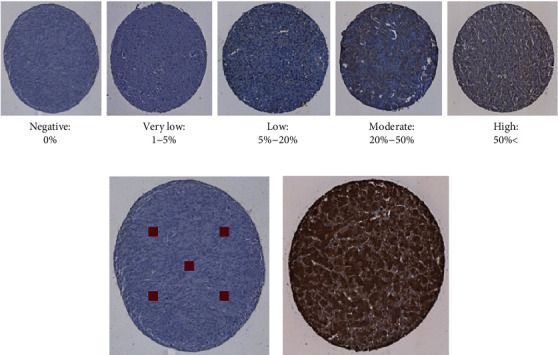
Scoring criteria for IHC. (a) Cores were scored as having negative (0%), very low (1-5%), low (5-20%), moderate (20-50%), or high (>50%) expression. (b) Squares represent areas where staining was assessed in each core. (c) Example of positive control tissue (liver).

Tissue slides were scanned using the Nanozoomer in the Nottingham University Hospital's Histopathology Department at ×40 magnification. Slides were then visualised using the Nanozoom Digital Pathology (NDP.zoom2) software programme. Cores were scored by two independent researchers for protein expression based on the criteria shown in Figure 1. The staining was classified as negative (0%), very low (1-5%), low (5-20%), moderate (20-50%), and high (>50%). There were three cores for each individual patient, where any disparity occurred between core scores; the average level of protein expression was recorded. An example of the different expression levels of the arginine pathway enzymes is illustrated in Supplementary figure 1.

### 2.3. Statistical Analysis

IHC scoring data was analysed using Microsoft Excel and figures generated as percentages of the total number of samples for each tumour type. Patient baseline characteristics were analysed in HGG, LGG, and medulloblastoma using SPSS, and the chi-squared test was used to look for any differences in individual antibody expression in HGG and LGG. It was also used to test for any associations between antibody expression and age or gender.

## 3. Results

Immunohistochemical analysis of arginine pathway enzymes was performed in a panel of children's TMAs with a range of histological subtypes. These included pHGG, LGG, MB, and EPN. The total number of individual cases (with replicates) stained using each specific antibody is detailed in the tables below (supplementary tables 1–4). The figures represent the percentage (normalised to 100%) of the different categories of IHC staining.

### 3.1. Paediatric High-Grade Glioma (pHGG)

**Figure 2:**
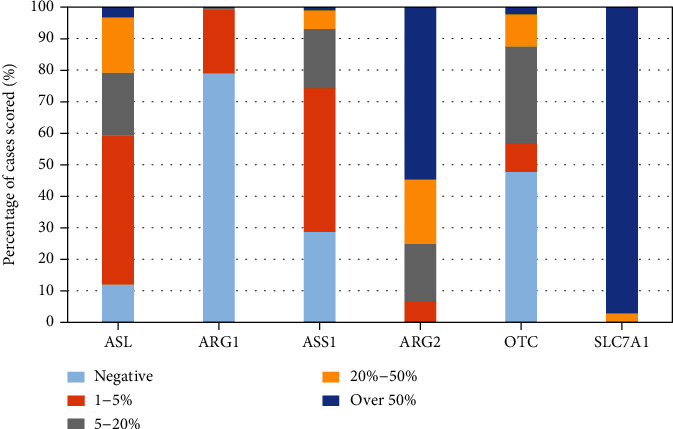
Expression of arginine pathway enzymes in pHGG expressed as a percentage normalised to 100% of the total number of cases scored per antibody. Tumours were classed as being deficient if the expression level of an individual pathway enzyme was 20% or lower. High expression was defined as >50% antibody staining (see supplementary table 1 for number of samples analysed per antibody in the pHGG cohort).

Almost all pHGG tumours showed high levels of expression (>50%) of the arginine transporter SLC7A1. Arg 2 was the main metaboliser expressed in moderate to high levels in 80% of cases. Arg 1 staining was negative or very low in all cases. The majority (87.5%) of tumours were OTC deficient, classed as having <20% expression. ASS1 was also similarly deficient in 94% of cases, and ASL was deficient in 79% of the cases (Figure 2, supplementary table 1).

### 3.2. Low-Grade Glioma (LGG)

**Figure 3:**
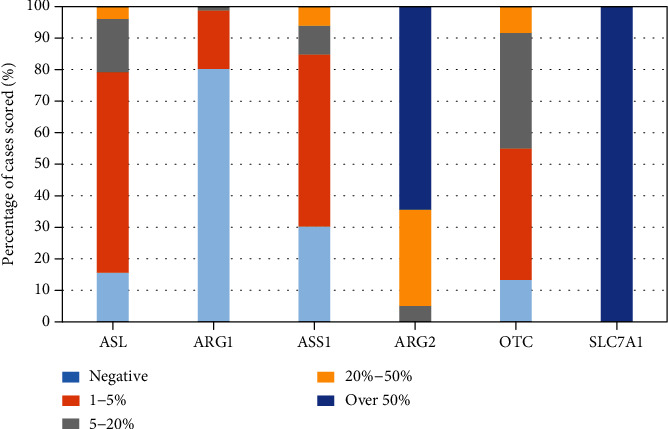
Expression of arginine pathway enzymes in LGG expressed as a percentage normalised to 100% of the total number of cases scored per antibody. Tumours were classed as being deficient if the expression level of an individual pathway enzyme was 20% or lower. High expression was defined as >50% antibody staining (see supplementary table 2 for number of samples analysed per antibody in the LGG cohort).

In the LGG samples analysed, 100% of cases showed high expression of SLC7A1. Arg2 was the main metaboliser in 94% of cases with Arg 1 being negative or very low level expression in 98% of cases. Recycling enzymes ASL, ASS, and OTC were deficient (<20% expression) in 96%, 93%, and 91% of cases (Figure 3, supplementary table 2).

### 3.3. Medulloblastoma

**Figure 4:**
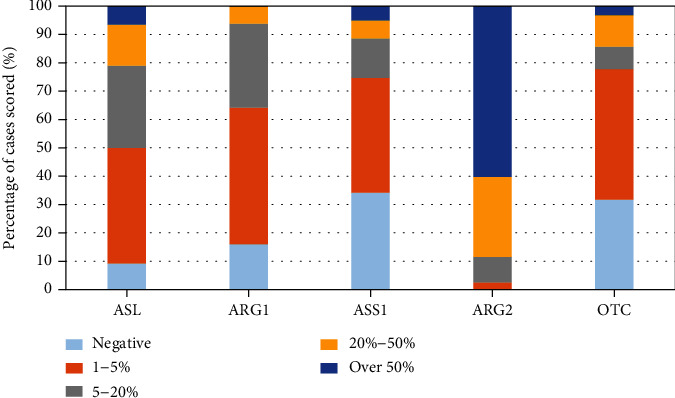
Expression of arginine pathway enzymes in MB expressed as a percentage normalised to 100% of the total number of cases scored per antibody. Tumours were classed as being deficient if the expression level of an individual pathway enzyme was 20% or lower. High expression was defined as >50% antibody staining (see supplementary table 3 for number of samples analysed per antibody in the MB cohort).

In MB cases, we focused on the metabolisers and the recycling enzymes. We found that Arg2 is the main metaboliser in 88% of MB tumours and that these were deficient in recycling enzymes ASL, ASS1, and OTC in 79%, 88%, and 85% of cases (Figure 4, supplementary table 3).

### 3.4. Ependymoma

**Figure 5:**
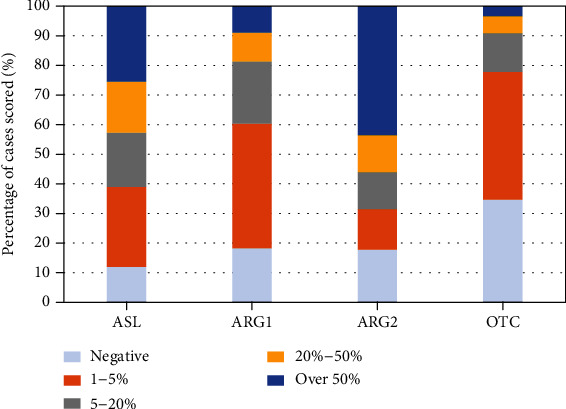
Expression of arginine pathway enzymes in EPN expressed as a percentage normalised to 100% of the total number of cases scored per antibody. Tumours were classed as being deficient if the expression level of an individual pathway enzyme was 20% or lower. High expression was defined as >50% antibody staining (see supplementary table 4 for number of samples analysed per antibody in the EPN cohort).

Similar to the previous results, Arg2 is the main metaboliser in EPN. However, expression of Arg 2 (>20%) was detected in 56% of cases. Arg1 was expressed as the metaboliser in 19% cases. Recycling enzymes ASL and OTC were deficient in 57% and 91% (Figure 5, supplementary table 4).

There were no significant difference levels in the expression levels of the individual enzymes between pHGG and LGG. There was no significant difference in expression of pathway enzymes based on age or gender across the different tumour groups analysed.

## 4. Discussion

These results present the first characterisation of arginine pathway enzyme expression in the most prevalent paediatric CNS tumours using immunohistochemical methods. The results suggest strongly that the majority of these tumours are likely to be arginine auxotrophs, albeit to varying degrees. They utilise arginine, as evidenced by the presence of the transporter SLC7A1 and the metaboliser Arg2. A significant proportion of the tumours have absent or low OTC, which suggests that they are unable to de novo synthesise arginine from ornithine and citrulline. This provides supporting evidence of selective metabolic vulnerability in these tumours that may be exploited therapeutically.

Additionally, our work demonstrates that we can use immunohistochemistry in FFPE tissue to screen patients for the arginine auxotrophy signature. This would be a simple, locally available tool to identify patients suitable for arginine depletion therapy in the future.

Moreover, our observation that a wide range of paediatric brain tumours has a similar metabolic vulnerability suggests that arginine dependency could be traced back to neural stem cells. This is consistent with published evidence in neuroblastoma where neural crest-derived primary cells are enriched for Arg2 rather than Arg1 [23]. It is also postulated that radial glial cells are the progenitor cells that are precursors of both ependymal cells and astrocytes [28, 29]. Medulloblastoma subtypes potentially have different cells of origin based on the key pathways that are altered, e.g., the Wnt/beta-catenin or sonic hedgehog (SHH) mutated tumours [30].

The TMAs we have analysed had not been annotated into molecular subgroups as per the new WHO classification into different subgroups. However, we have analysed a large number of samples in each tumour type and the auxotrophy signature suggests that the requirement for arginine could potentially be traced back to an early precursor cell and may not necessarily vary based on subgroup. However, further work is needed to confirm this.

Studies have shown that BCT-100 can deplete serum arginine effectively. The ability of the drug to cross the blood–brain barrier (BBB) is unknown. However, it is currently being used to treat glioblastoma multiforme in adult and paediatric clinical trials. Moreover, clinical trials in adults have shown very few side effects. There is, however, potential for extracellular arginine depletion to reduce the amount of arginine that is available to cross the BBB. Therefore, arginine depletion provides a potential solution of overcoming the challenge of drug delivery across the BBB.

Further work is required in cell culture and animal models to study the effect of arginine depletion in these groups of tumours. If arginase treatment intravenously can deplete arginine in the CNS or the CSF and can cause cytotoxic or cytostatic effects on the tumour, this would be an exciting therapeutic target. Arginine is also a source of polyamine synthesis, and this has been demonstrated to play a key role in promoting tumour growth and cell proliferation in a number of cancers including medulloblastoma [31, 32]. Therefore there may be potential for synergy with polyamine synthesis inhibitors such as difluoromethylornithine (DFMO) that are currently in early phase trials in children with medulloblastoma (NCT04696029).

The first paediatric trial of arginine depletion using BCT-100 is ongoing, and the results will clearly be informative. However, this trial includes only paediatric HGG patients, whilst other CNS tumours are not currently included. Once the safety of the drug has been established in the paediatric age group, it would be interesting to study the efficacy of arginine depletion in all the tumour groups detailed.

## 5. Conclusion

The majority of paediatric CNS tumours, namely, medulloblastoma, ependymoma, high-grade glioma, and low-grade glioma, are likely arginine auxotrophs. Extracellular arginine depletion could potentially be used to treat these patients, and this is currently under study, in which case immunohistochemistry of FFPE tissue could be used to identify patients who are likely to benefit from this treatment.

## Figures and Tables

**Table 1:** List of antibodies used for the different tumour types.

Tumour type	Antibodies used for immunohistochemistry					
	ASL	ARG1	ASS1	ARG2	OTC	SLC7A1
HGG	x	x	x	x	x	x
LGG	x	x	x	x	x	x
MB	x	x	x	x	x	
EPN	x	x		x	x	

## Data Availability

Data is available on request from the authors.

## Supplementary Materials

Supplementary Materials Supplementary Figure 1: examples of antibody expression for the recycling enzymes, OTC, ASS1, and ASL (blue); the catabolic enzymes, Arg1 and Arg2 (red); and arginine transporter, SLC7A1 (black) in pHGG. Positive immunostaining (brown staining) with nuclei stained blue by haematoxylin. The red triangles highlight these areas of expression. All images were taken at magnification ×40. Supplementary Table 1: pHGG cases scored. Supplementary Table 2: LGG cases scored. Supplementary Table 3: MB cases scored. Supplementary Table 4: EPN cases scored.
