# Supplementary material for: Characterisation of Expression the Arginine Pathway Enzymes in Childhood Brain Tumours to Determine Susceptibility to Therapeutic Arginine Depletion
Source: Biomed Res Int. 2022 Jun 22;2022:9008685. doi: 10.1155/2022/9008685 (PMC9242779; doi:10.1155/2022/9008685)
Supplement: Supplementary Materials — Supplementary Figure 1: examples of antibody expression for the recycling enzymes, OTC, ASS1, and ASL (blue); the catabolic enzymes, Arg1 and Arg2 (red); and arginine transporter, SLC7A1 (black) in pHGG. Positive immunostaining (brown staining) with nuclei stained blue by haematoxylin. The red triangles highlight these areas of expression. All images were taken at magnification ×40. Supplementary Table 1: pHGG cases scored. Supplementary Table 2: LGG cases scored. Supplementary Table 3: MB cases scored. Supplementary Table 4: EPN cases scored. [file 9008685.f1.docx]

**
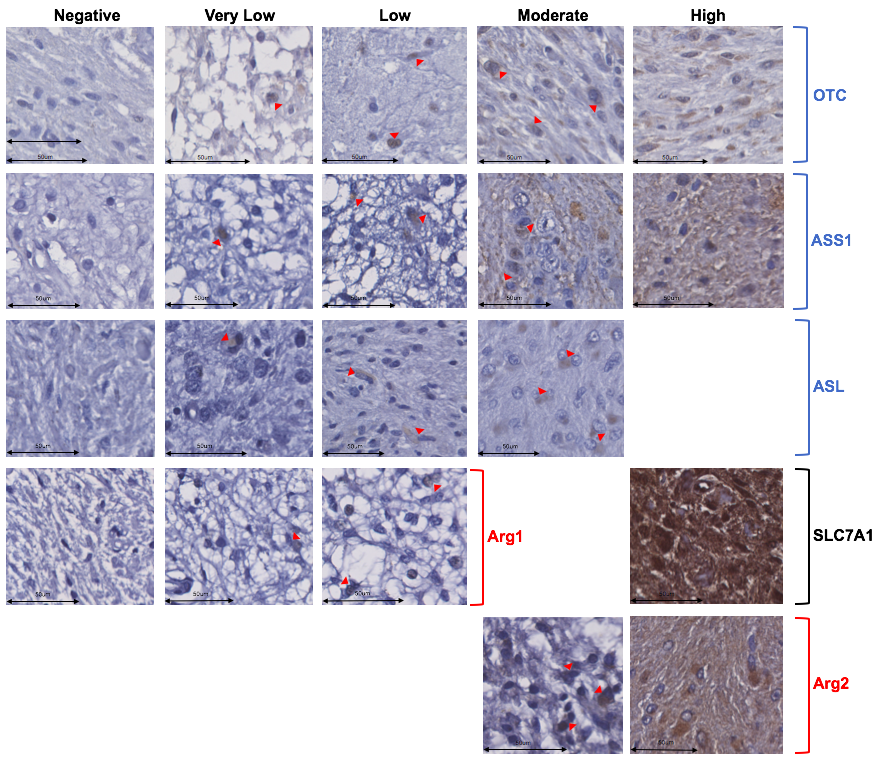
Supplementary figure 1**: Examples of antibody expression for the recycling enzymes, OTC, ASS1 and ASL (blue), the catabolic enzymes, Arg1 and Arg2 (red) and arginine transporter, SLC7A1 (black) in pHGG. Positive immunostaining (brown staining) with nuclei stained blue by haematoxylin. The red triangles highlight these areas of expression. All images were taken at magnification x40

Supplementary table 1: pHGG cases scored

| Scoring system |  | Number of cases scored for each antibody | | | | | |
| --- | --- | --- | --- | --- | --- | --- | --- |
|  |  | ASL | ARG1 | ASS1 | ARG2 | OTC | SLC7A1 |
| Negative |  | 15 | 98 | 29 | 0 | 42 | 0 |
| 1-5% |  | 59 | 25 | 46 | 7 | 8 | 0 |
| 5-20% |  | 25 | 1 | 19 | 20 | 27 | 0 |
| 20%-50% |  | 22 | 0 | 6 | 22 | 9 | 3 |
| Over 50% |  | 4 | 0 | 1 | 59 | 2 | 99 |
| Total |  | 125 | 124 | 101 | 101 | 88 | 102 |

Supplementary table 2: LGG cases scored

| Scoring system |  | Number of cases scored for each antibody | | | | | |
| --- | --- | --- | --- | --- | --- | --- | --- |
|  |  | ASL | ARG1 | ASS1 | ARG2 | OTC | SLC7A1 |
| Negative |  | 12 | 65 | 10 | 0 | 8 | 0 |
| 1-5% |  | 49 | 15 | 18 | 0 | 25 | 0 |
| 5-20% |  | 13 | 1 | 3 | 3 | 22 | 0 |
| 20%-50% |  | 3 | 0 | 2 | 18 | 5 | 0 |
| Over 50% |  | 0 | 0 | 0 | 38 | 0 | 43 |
| Total |  | 77 | 81 | 33 | 59 | 60 | 43 |

.

Supplementary table 3: MB cases scored

| Scoring system |  | Number of cases scored for each antibody | | | | |
| --- | --- | --- | --- | --- | --- | --- |
|  |  | ASL | ARG1 | ASS1 | ARG2 | OTC |
| Negative |  | 7 | 13 | 27 | 0 | 20 |
| 1-5% |  | 31 | 39 | 32 | 2 | 29 |
| 5-20% |  | 22 | 24 | 11 | 7 | 5 |
| 20%-50% |  | 11 | 5 | 5 | 22 | 7 |
| Over 50% |  | 5 | 0 | 4 | 47 | 2 |
| Total |  | 76 | 81 | 79 | 78 | 63 |

Supplementary table 4: EPN cases scored

| Scoring system |  | Number of cases per score for each antibody | | | |
| --- | --- | --- | --- | --- | --- |
|  |  | ASL | ARG1 | ARG2 | OTC |
| Negative |  | 32 | 47 | 47 | 114 |
| 1-5% |  | 72 | 108 | 36 | 142 |
| 5-20% |  | 49 | 54 | 33 | 43 |
| 20%-50% |  | 46 | 25 | 33 | 19 |
| Over 50% |  | 68 | 23 | 115 | 11 |
| Total |  | 267 | 257 | 264 | 329 |
